# Insights into the neural mechanisms underlying hand praxis: implications for the neurocognitive rehabilitation of apraxia

**DOI:** 10.3389/fpsyg.2014.01380

**Published:** 2014-12-09

**Authors:** Jorge Oliveira, Rodrigo Brito

**Affiliations:** COPELABS, Universidade Lusófona de Humanidades e TecnologiasLisbon, Portugal

**Keywords:** cognitive rehabilitation, apraxia, priming, praxis, cerebral dominance, transitive gestures, intransitive gestures

The understanding of the neural basis of limb apraxia (deficits in performing previously learned skilled movements) has benefited greatly from cognitive research on gesture processing and recognition, but the influence of this research on the treatment of apraxia is limited (Cantagallo et al., 2012). In our view, it would be desirable that this field of research have an impact also on treatment of apraxia, and Helon and Kroliczak's (2014) studies offer useful insights for the design of apraxia treatments.

Apraxia typically results from left-brain lesions but impairs movements in both upper limbs equally. This is because disturbances affect the conceptual formulation of the movement rather than their executive ability (Frey, 2008). Research has also shown that access to internal representations underlying tool-use pantomimes (transitive) is more likely to be impaired than access to internal representations of communicative (intransitive) gestures (Foundas et al., 1999). The most common explanation is that tool-use actions are more left-lateralized than communicative gestures (Stamenova et al., 2010), but recent neuroimaging studies (Króliczak and Frey, 2009; Kroìliczak, 2013) suggest a common representational system, on which transitive gestures are however more dependent. Helon and Kroliczak's (2014) finding that categorization of transitive and intransitive gestures were affected by both pictorial and linguistic priming in the right-visual field (RVF) suggests that communicative and tool-use actions share a left-lateralized mechanism intersecting language functions.

This converges with results from the recent literature on the cognitive rehabilitation of limb apraxia (for reviews, see Buxbaum et al., 2008; Cantagallo et al., 2012), which includes treatments focusing on alleviating apraxic deficits by relearning motor sequences, and treatments designed to compensate for apraxic deficits by improving the functioning of processes spared by apraxia. In both approaches, the use of contextual and object-related cues can improve procedural memory, which may explain why patients are more proficient in reproducing gestures in their natural context than in a laboratory or clinical setting. This relies on priming and, because priming is somewhat resistant to brain damage, priming-based rehabilitation is one of the techniques of choice to restore hand praxis skills (Sohlberg and Mateer, 2001).

Priming refers to a change in the nervous system triggered by prior exposure to a stimulus, which guides the order of activation in a given neural network according to previous experience (Pachalska et al., 2002). Given the dichotomy of privileged verbal left-hemisphere processing vs. visual right-hemisphere processing, Helon and Kroliczak (2014) had participants carry out a behavioral task to test whether the effects of pictorial (study 1) and linguistic (study 2) primes biased either to the left (RVF) or right hemisphere (left visual field) would affect categorization of clips of either meaningful transitive, meaningful intransitive, or meaningless hand movements. They found that intransitive gestures were categorized faster in both studies (pictorial and linguistic priming), which indicates lower complexity. They also found that categorization of both types of gestures was facilitated by RVF presentation of pictorial primes, but this was stronger for transitive gestures, whereas linguistic priming effects following RVF presentation were significant only for intransitive gestures.

At a basic level, the common effects of RVF priming for both gestures, consistent with recent findings with fMRI (Króliczak and Frey, 2009; Kroìliczak, 2013) and with known deficits in both language and praxis skills following left-brain damage, suggest a common neural substrate underlying language and praxis. This supports the idea that complex tool-use skills were evolutionary precursors of both non-verbal communication and language functions (Frey, 2008). In fact, there is evidence that praxis skills are more associated with the laterality of language functions than with hand preference (Meador et al., 1999). Recent neuroimaging data (Kroliczak et al., 2011) provide further evidence for an association between praxis and language by showing that this relationship remains even in left-handers with atypical lateralization of language.

What do these results suggest for rehabilitation? First, more attention should be paid to the interdependence between motor and language functions when dealing with apraxic patients. “Intersystemic gestural reorganization” (Luria, 1970), a method that uses gestures to improve verbal production in aphasic patients, could provide a model to integrate those functions: an intriguing but untested possibility is that the reverse treatment (train verbal descriptions of gestures) could improve gestural performance in apraxia patients.

Additionally, the deficits that apraxic patients often exhibit in gestural communication (Borod et al., 1989) point to its importance in the development of rehabilitative treatments. Smania et al. (2000, 2006) have shown that training both transitive and intransitive gestures, using contextual information (pictures depicting objects) produces significant improvements in specific apraxia tests and functionality. Considering the links between language and action, it seems probable that the use of pictorial cues depicting an object or context, combined with linguistic cues of words describing the same situations, will prompt the involvement of different neural pathways in accessing stored action representations, thus maximizing the probability of success of rehabilitative treatment. Involving both cues in pilot treatment programmes need not wait for further research to better clarify their roles (note that effects of verbal commands could be different from visual cues and need to be studied).

Second, the differential effects found by Helon and Kroliczak (2014) for transitive and intransitive gestures support a greater complexity account of tool-use pantomimes over communicative gestures (Carmo and Rumiati, 2009), which from a practical viewpoint should be considered when developing training-based rehabilitative treatments for apraxia. One possible approach to these treatments could rely on mixed-reality systems that are under development for motor rehabilitation, such as augmented-reality environments that allow users to view a representation of their own body interacting with the real world (Regenbrecht et al., 2012). It is possible and desirable that these features will provide a standard for rehabilitation in the near future, and that this may not be limited to motor training, but may also be used to train either planning or execution components of the motor system involved in transitive and intransitive actions.

## Conflict of interest statement

The authors declare that the research was conducted in the absence of any commercial or financial relationships that could be construed as a potential conflict of interest.
